# Hospital Progress and Finance

**Published:** 1924-10

**Authors:** 


### HOSPITAL PROGRESS AND FINANCE.

### GUY'S BICENTENARY.

The preparation for the great appeal to be made in connection with the bicentenary of Guy's Hospital in 1925 is well advanced. The sum needed is £200,000, of which £40,000 is for the medical school. Only once before, in 1896, has the hospital made a general appeal of this kind. The campaign begins in October, and will not be allowed to flag before Christmas, since January 6 is the anniversary of the opening of the hospital.

### THINKING IN COUNTIES.

The inquiry into the number of hospital beds available or needed is producing interesting results, and, as was to be expected, the figures obtained from the different areas are much more impressive than those from single hospitals have been in the past. A typical instance is that of Staffordshire. The return shows that the county possesses nine general and eight cottage hospitals. The three hospitals principally in need of extension are the North Staffordshire Hospital, the Wolverhampton and Staffordshire, and the Walsall General Hospital. These three can now accommodate 614 patients. But they need 545 more beds. The hospital position of one county is thus vividly revealed. Now that we are beginning to think in districts and areas, the defects of our hospital system are more likely to be remedied. Everything that modifies the old isolated self-sufficiency of hospitals in favour of co-ordination is to the good, and the current is setting steadily in this direction.

### BETTER NEWS FROM KING'S.

It is very satisfactory to note the improved financial position of King's College Hospital. An anonymous donor has cancelled a loan of £20,000, and this with the special grant of £16,000 from King Edward's Hospital Fund has reduced the debt to £37,000. A gratifying result is the reopening of fourteen more beds for cases of accident. Annual subscribers continue to grow in number, and we trust that the worst of this hospital's financial troubles is now past.

### A TROUBLESOME BEQUEST.

The centenary of Swansea Hospital has led Mr. David Salmon to write an historical sketch of the institution which contains a curious story. The hospital was successfully started by a legacy that was never paid fully, and a gift in kind that was handed over put the hospital to some expense. The first resident surgeon, Dr. Edwards, in 1831 left the hospital his library and £1,500 to be paid on the death of his wife. The announcement made the launching of the scheme unexpectedly successful, but it was later discovered that the doctor had entered into a bond with two other men for over £4,000, and as they proved to have no property, the bulk of the money had to be found from the doctor's estate. After nine years of negotiations the hospital received £335 and the library, but the library cost £122 to store until space could be found for it. An odd point in the story is that the reason why Dr. Edwards had entered into this bond has not been discovered. Hospital histories are full of curious occurrences, and we are always glad when they find historians. The life of a hospital is in its traditions, and without a history many are lost.

### AN OBJECT LESSON FOR TESTATORS.

The decision of King Edward's Hospital Fund to apply the legacies of £200,000 left by the late Mr. and Mrs. John Wells to aid the London hospitals with extensions and improvements is very welcome. Its first result has been the opening of fourteen beds for accidents at King's College Hospital. Despite the grants made in the past for special purposes and the emergency distribution made after the war, the Fund has necessarily had to provide first of all for the hospitals' maintenance. Without its help, the post-war crisis might not have been surmounted. Now that the pressing difficulty is the insufficiency of beds, the Fund is able to reserve some of its resources for grants toward capital expenditure. There could be no better instance of its power and flexibility, or of the ease with which a voluntary, as opposed to a public, authority can adapt itself to the changing necessities of the moment. These facts are not lost upon testators, as their steady support of King Edward's Fund happily proves.

### TUBERCULOUS CHILDREN AND GENERAL HOSPITALS.

In an interesting speech at a recent meeting of the Corbett Hospital, Stourbridge, of which he is president, Viscount Cobham called attention to the tuberculous children who came to the hospital, and lamented how little at present could be done for them there. He said that he would like to see this side of the work developed, and, indeed, it raises an important question. The Corbett Hospital is small, and has little to spare when what may be called its regular necessities are met. But these children, it seems, continue to come, and yet can be benefited very little. What is the solution ? The matter cannot remain where it is, but why should nothing be done until the hospital itself can provide some at least of the appropriate treatment? It has a responsibility for these children, and one way of meeting this for the time being would be to get into touch with the orthopædic institutions and clinics that are now springing up, particularly in Shropshire. They may, indeed, have their own limited field, but co-operation between institutions is the order of the day, and some arrangement should be possible. At all events, an attempt ought to be made, for to receive cases is to accept responsibility for them which the provision of a bed does not fulfil.

### A NEW CHAPTER IN WALES.

The first meeting of the reconstituted body of governors of the King Edward VII. Welsh National Memorial Association, recently held, ended an interesting chapter in its history. With 1,400 beds at its disposal, the Association, now that the hospital at Cefn Mably has been opened, has completed the scheme originally planned. In an interesting retrospect Mr. David Davies, M.P., has explained how the capital cost of sixteen hospitals and sanatoria has been found by the Association without a farthing from the rates. The capital of the Association, however, will not last for ever. Indeed the completion of the works now sanctioned will exhaust the remainder, when the Association will have to consider how fresh capital can be secured. Mr. Davies was able to state that, though 260 patients had been waiting admission to the sanatoria, the medical officers believe that no early case is included. By the opening of Cefn Mably the number of pulmonary patients awaiting hospital treatment had been reduced to 63. The idea is, of course, to make complete provision, and so to organise preventive work that the Association controls the disease, not the disease the Association. Arrears have almost been caught up. The new chapter begins with this accomplished.

### "THE CRIPPLES' JOURNAL."

A proved test of the probable value of new journals is the definiteness or vagueness of the ends which they propose to maintain; for if editors have not a definite policy before they start, they do not often find one later. "The Cripples' Journal," as we have briefly noted, is a new quarterly published, price one shilling, by the Shropshire Orthopædic Hospital, Oswestry, and it survives this test. It intends to publish technical articles on orthopædics by authorities, so that all developments of treatment may be known; to recount in turn the work of well-known hospitals, including those abroad, and to be a means of propaganda. To be extended and supported, the work of these hospitals and after-care centres must be known, and it is to their advantage to have a common fund of information in a separate paper. It happens that Shropshire is a county which has been early in the organisation of hospitals and after-care centres for cripples; thus the Journal springs from an already active district. The second number appears this month. No sportsman could have read without interest Dr. McCrae Aitken's article on "Gymnastics and Orthopædic Treatment" in the first issue, and the account of the sunlight treatment at Leysin will be read eagerly by any layman intelligent enough to be familiar with Dr. Rollier's name. We mention the layman because he is growing alive to the fascination of orthopædics. For the orthopædic worker such articles will be irresistible. The Journal, which also contains news from the different centres, could hardly have made a more promising start.

### THE MORAL OF BRADFORD.

The Bradford City Council has decided not to adopt the proposal of its Finance Committee to make an annual grant equivalent to a penny rate for five years to the new Infirmary Fund. This, as we noted last month, was the proposal that was made when the original recommendation to purchase the site and buildings of the Infirmary had twice been referred back. The reason for this decision is, in effect, that the municipality has now its own hospital in St. Luke's, and it is felt that municipal money should go to municipal institutions. It is apparent that such arguments would not have been used were the City Council still prepared to purchase the Infirmary buildings. But they arose inevitably when this plan was abandoned, and the alternative of a grant to the Infirmary, in return for representation, was substituted. In sum, a municipality is not inclined to support both municipal and voluntary hospitals. The decision will disappoint many friends of the Royal Infirmary, but if it leads them to resolve to be independent of municipal support and to concentrate their efforts on raising the necessary money from voluntary sources, then the voluntary system may be strengthened in the long run. The war and altered circumstances have rendered the sale of the Infirmary buildings to the Corporation impossible, and that appears to be the only form of help which the two can judiciously render to each other. Let them now, therefore, pursue, as before, parallel but independent ways.

### THE ALMONERS' CONFERENCE.

The recent conference of the Hospital Almoners' Association, which was the first of its kind, marks the importance which this department is now recognised to play in hospital life. The immediate object was to provide an opportunity for discussion between Almoners in different parts of the country, and, since social conditions so largely enter the problems with which the Almoner has to deal, the value of this is obvious. It was a curious comment on the change with which the Almoner's work has come to be regarded that the addresses at the conference were given by medical men. It seems but the other day when Almoners were expected chiefly to check the abuse of the out-patient department, and when after-care was limited to a farewell gift from the Samaritan fund. Happily, even now, when Almoners have also to assess a patient's capacity to contribute, they are mainly dispensers, not collectors, of assistance, and the hospital social service unit is not so far off as one of the speakers lamented. At future conferences we hope that Almoners also will deliver addresses. Their profession has only begun to develop its possibilities, and we congratulate the Association on the new departure, which should now become an annual event. No side of hospital work is growing more rapidly than the Almoner's.

### PROGRESS OF BRITISH DENTISTS' HOSPITAL.

The British Dentists' Hospital, founded in 1911, with headquarters at Camden Road, N.W. 1, and branches at Lewisham Park and Clapham Common, continues to grow as more and more people make use of it. Not only are additional sessions being held, but the committee is improving the clinics opened during the war and controlled by arrangement with the borough councils. Medical officers of health recommend patients from their districts, and the welfare workers secure a continuous supply. The fees are moderate, and there is no teaching or students.

